# The Association of *MEG3* Gene rs7158663 Polymorphism With Cancer Susceptibility

**DOI:** 10.3389/fonc.2021.796774

**Published:** 2021-12-09

**Authors:** Xueren Gao, Xianyang Li, Shulong Zhang, Xiaoting Wang

**Affiliations:** ^1^ School of Pharmacy, Yancheng Teachers’ University, Yancheng, China; ^2^ Department of General Surgery, Xuhui District Central Hospital, Shanghai, China; ^3^ Physical Examination Centre, Xuhui District Central Hospital, Shanghai, China

**Keywords:** MEG3, polymorphism, rs7158663, cancer, susceptibility

## Abstract

Although the association of *MEG3* gene rs7158663 polymorphism with cancer susceptibility has been investigated, the findings are inconsistent. The aim of this study was to analyze the association between the rs7158663 polymorphism and cancer susceptibility through a case-control study and meta-analysis. In a case-control study with 430 colorectal cancer (CRC) cases and 445 healthy controls, the rs7158663 polymorphism was genotyped by direct sequencing. STATA software was used to calculate the pooled odds ratio and 95% confidence interval in a meta-analysis including 4,649 cancer cases and 5,590 controls. Both the case-control study and meta-analysis showed that the rs7158663 polymorphism was associated with increased susceptibility to CRC. Individuals carrying the AA or GA genotype were more likely to develop CRC than those carrying the rs7158663 GG genotype. Interestingly, MEG3 expression was significantly lower in colorectal tissues of the AA or GA genotype compared to those of the rs7158663 GG genotype. In addition, the meta-analysis suggested that the rs7158663 polymorphism was also associated with increased susceptibility to breast cancer and gastric cancer. Bioinformatics analysis showed that the rs7158663 A allele contributed to the binding of hsa-miR-4307 and hsa-miR-1265 to MEG3. In conclusion, the current findings suggest that the *MEG3* gene rs7158663 polymorphism may serve as a genetic marker for predicting the risk of cancers, such as breast cancer, gastric cancer and CRC. However, the sample size of the current study is still insufficient, especially in the subgroup analysis. Therefore large and well-designed studies are needed to validate our findings.

## Introduction

Cancer is one of the most serious public health issues in the world, with approximately 18.1 million new cancer diagnoses and 9.6 million cancer deaths in 2018 (1). Although the precise processes of cancer development and progression are still largely unclear, a growing body of research suggests that genetic predisposition has a substantial influence on the likelihood of individual cancer development (2, 3).

Long non-coding RNA (lncRNA) is a form of RNA transcript that is longer than 200 nucleotides but does not transcribe into protein in cells. LncRNAs play a role in a variety of cell activities, such as cell proliferation, migration, invasion, and angiogenesis, and their dysregulation has been linked to a variety of cancers (4–7). Maternally expressed 3 (MEG3) is one of the most well-studied lncRNAs and is expressed in multiple organs, such as the liver, brain, pancreas, stomach and ovary. However, MEG3 expression is typically suppressed in a variety of cancer tissues (8). Functional studies showed that this lncRNA could regulate the expression of various tumor suppressor genes and oncogenes (9–12). Zhu et al. found that ectopic expression of MEG3 could significantly inhibit proliferation and induce apoptosis in hepatoma cells. MEG3 could function as a tumor suppressor in hepatoma cells through interacting with p53 protein to activate p53-mediated transcriptional activity and influence the expression of partial p53 target genes (9). Dong et al. found that downregulation of MEG3 expression could promote proliferation, migration, and invasion of hepatocellular carcinoma cells by upregulating TGF-β1 expression (10). Zuo et al. demonstrated that MEG3 activated by vitamin D could inhibit glycolysis in colorectal cancer (CRC) *via* promoting c-Myc degradation (11). Xu et al. found that MEG3 could mediate the miR-149-3p/FOXP3 axis by reducing p53 ubiquitination to exert a suppressive effect on regulatory T cell differentiation and immune escape in esophageal cancer (12). In addition, certain polymorphisms (rs3087918 T>G, rs11160608 A>C, rs4081134 G>A, rs7158663 A>G) within the *MEG3* gene are implicated in cancer susceptibility (13–16). For example, *MEG3* gene rs3087918 was associated with a decreased risk of breast cancer in a Chinese population (14). *MEG3* gene rs11160608 was related to an increased risk of oral squamous cell carcinoma in a Chinese Han population (15). *MEG3* gene rs4081134 was significantly associated with a decreased risk of lung cancer in a Northeast Chinese population (16). *MEG3* gene rs7158663 is the most interesting polymorphic locus located on the MEG3 transcript. Bioinformatic analysis showed that the rs7158663 polymorphism had the potential to change the local RNA folding structure and affect miRNA-lncRNA interactions, which in turn affected the expression level of miRNA and/or MEG3 (17, 18). Several studies have explored the relationship between this potentially functional polymorphism and cancer susceptibility, but the results are inconsistent and need to be further clarified.

In the current study, we first explored the relationship between the rs7158663 polymorphism and CRC susceptibility using a case-control study, and then analyzed its effect on MEG3 expression in colorectal tissues. In addition, a meta-analysis was conducted to systematically evaluated the relationship between this polymorphism and cancer susceptibility, which would help us to better understand the role of the rs7158663 polymorphism in cancer susceptibility.

## Materials And Methods

### Sample Collection

Peripheral blood of 430 CRC patients and 445 healthy controls were collected from Shanghai Xuhui District Central Hospital. All participants were genetically unrelated Han Chinese. Diagnosis of CRC patients was histopathologically confirmed. Healthy controls were cancer-free individuals living in the same residential area and seeking routine physical exams. Furthermore, colorectal tissues were obtained from 40 CRC surgery patients who had not received radiochemotherapy before surgery. Written informed consent was obtained from all participants. The experimental protocol was established, according to the ethical guidelines of the Helsinki Declaration and was approved by the Human Ethics Committee of Shanghai Xuhui District Central Hospital.

### Genotyping

TIANamp genomic DNA Kit (Tiangen) was used to isolate genomic DNA from peripheral blood according to the manufacturer’s instructions. Genomic DNA concentration was detected using a NanoDrop spectrophotometer. Direct sequencing was used to detect the genotype of the rs7158663 locus in each individual.

### Real-Time Quantitative PCR

Total RNA was isolated from colorectal tissues using the RNAsimple total RNA kit (Tiangen) according to the manufacturer’s instructions. ReverTra Ace qPCR RT Kit (TOYOBO) was used to synthesize cDNA. FastStart Universal SYBR Green Master (Roche) was used to conduct real-time quantitative PCR. MEG3 expression was normalized to the internal control GAPDH. The specific primer sequences are presented in **Table S1**.

### Bioinformatic Analysis

The lncRNASNP online tool (http://bioinfo.life.hust.edu.cn/lncRNASNP) was used to analyze whether the rs7158663 polymorphism affects miRNA binding (19).

### Statistical Analysis

Hardy-Weinberg equilibrium (HWE) for the control group was tested by a goodness-of-fit χ2 test. The association of *MEG3* gene rs7158663 polymorphism with CRC susceptibility was evaluated using adjusted odds ratios (ORs) with their 95% confidence intervals (CIs). Student’s t-test was used to check the differences for age variable between CRC cases and controls. χ2 test was used to assess the differences in gender variable between CRC cases and controls. The normalized expression levels of MEG3 among different genotypes were compared using one-way ANOVA. All statistical analyses were performed by SAS 9.4 (SAS Institute, Cary, USA). P < 0.05 was defined as the level of significance.

### Meta-Analysis

PubMed, CNKI and EMBASE databases were searched based on the following keywords: “Maternally expressed 3 or MEG3”, “polymorphism or variant” and “cancer or carcinoma or malignancy”. The last literature search was conducted on October 7, 2021. The primary inclusion criterion for previous studies was to have sufficient genotype data. If numerous studies had overlapping or duplicate data, only studies with complete data were included. Data from the included studies were extracted independently by two investigators. Disagreements were settled by conversation. The pooled ORs and their 95% CIs were applied to determine the relationship of the rs7158663 polymorphism with cancer susceptibility. The between-study heterogeneity was assessed using Chi-square-based statistic I^2^ test and Cochran’s Q-test. When I^2^ > 50% or P_H_ < 0.1, we used the random-effects model to estimate the pooled OR. Otherwise, the fixed-effects model was applied. To assess the quality and consistency of the results, sensitivity analysis was undertaken by removing each study in turn. Begg’s and Egger’s tests were used to assess potential publication bias. Trial sequential analysis (TSA) was conducted in a selected genetic model to assess the statistical reliability of the meta-analysis. TSA was conducted with a 5% risk of type I error and a 20% risk of type II error (20). The statistical analyses were performed by STATA 12.0 (Stata Corporation, College Station, TX, USA).

## Results

The results of the case-control study are shown in **Table 1**. There was no statistical difference in the age and gender distribution between the case and control groups (P>0.05). The genotype frequency distribution of the control group was consistent with HWE (P_HWE_=0.43). There was a significant association between *MEG3* gene rs7158663 polymorphism and CRC susceptibility [GA vs. GG: OR=1.48, 95%CI= 1.11-1.96, P=0.007; AA vs. GG: OR=1.83, 95%CI=1.11-3.03, P=0.018; (GA+AA) vs. GG: OR=1.53, 95%CI=1.17-2.00, P=0.002; A vs. G: OR=1.41, 95%CI=1.14-1.74, P=0.001].

**Table 1:** Characteristics of age, gender and rs7158663 polymorphism in cases and controls.

Variables	Case (%) (N = 430)	Controls (%) (N = 445)	^a^OR (95% CI)	^a^P value
Age, mean ± SD	57.7 ± 5.9	57.7 ± 6.1		0.86
Gender				
Male	251 (58.4)	249 (56.0)		0.47
Female	179 (41.6)	196 (44.0)		
Genotype				
GG	202 (47.0)	256 (57.5)	Reference	
GA	185 (43.0)	159 (35.7)	1.48 (1.11-1.96)	0.007
AA	43 (10.0)	30 (6.7)	1.83 (1.11-3.03)	0.018
P_trend_				0.002
P_HWE_				0.43
GG	202 (47.0)	256 (57.5)	Reference	
GA+AA	228 (53.0)	189 (42.5)	1.53 (1.17-2.00)	0.002
GG+GA	387 (90.0)	415 (93.3)	Reference	
AA	43 (10.0)	30 (6.7)	1.55 (0.95-2.52)	0.08
Allele				
G	589 (68.5)	671 (75.4)	Reference	
A	271 (31.5)	219 (24.6)	1.41 (1.14-1.74)	0.001

a Adjusted for age and gender when appropriate.

**Figure 1:**
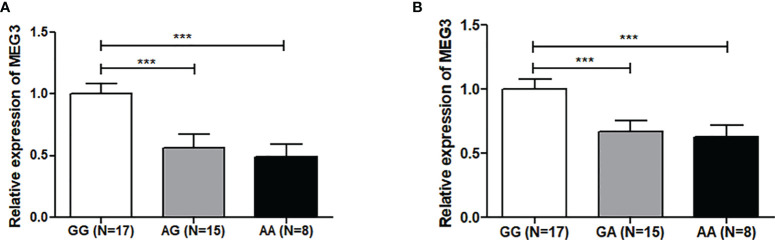
Relationship between rs7158663 genotype and MEG3 expression in CRC tissues **(A)** and normal paracancerous tissues **(B)**. ***p < 0.001.

**Figure 2:**
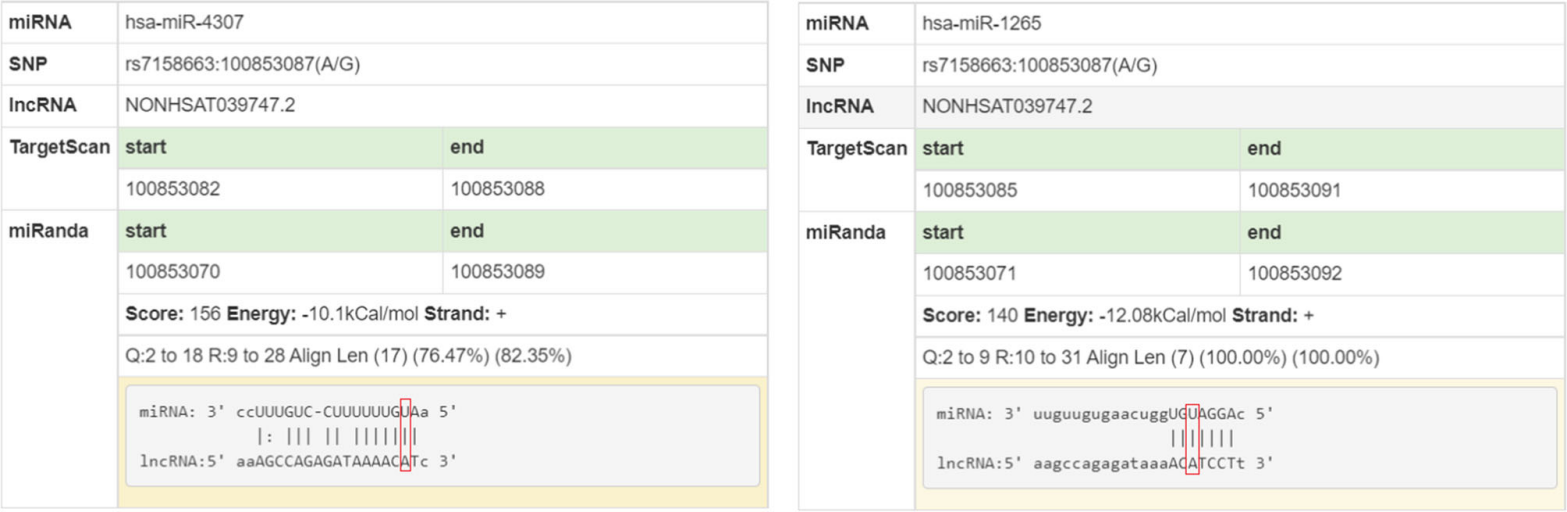
lncRNASNP-based analysis of the effect of rs7158663 polymorphism on miRNA binding.

Genotype-tissue expression results showed that the rs7158663 polymorphism was significantly associated with the expression of MEG3 in colorectal tissues. MEG3 expression was significantly lower in colorectal tissues of the AA or GA genotype compared to those of the rs7158663 GG genotype (**Figure 1**). The results of bioinformatics analysis showed that the rs7158663 A allele contributed to the binding of hsa-miR-4307 and hsa-miR-1265 to MEG3 (**Figure 2**).

Based on database searches, a total of 11 case-control studies exploring the association of the rs7158663 polymorphism with cancer susceptibility were included in the current meta-analysis (**Figure S1**). The relevant studies were published between 2016 and 2021. The current meta-analysis combined our results included 4,649 cancer cases and 5,590 controls (**Table 2**). The overall combined analysis showed that the rs7158663 polymorphism was not associated with cancer susceptibility (**Table 3**). However, the country-based stratified analysis showed that the rs7158663 polymorphism was associated with cancer susceptibility in the Chinese population under (AA+AG) vs. GG, AA vs. (AG+GG), AA vs. GG, AG vs. GG, and A vs. G models, and in the Egyptians under (AA+AG) vs. GG model. The stratified analysis based on cancer type showed that the rs7158663 polymorphism was associated with susceptibility to breast cancer under (AA+AG) vs. GG and AG vs. GG models, and gastric cancer under (AA+AG) vs. GG, AA vs. (AG+GG), AA vs. GG, AG vs. GG, and A vs. G models, and colorectal cancer under (AA+AG) vs. GG, AA vs. (AG+GG), AA vs. GG, AG vs. GG, and A vs. G models.

**Table 2:** Main characteristics of the case-control studies in the current meta-analysis.

Authors	Year of publication	Country	Cancer type	Genotyping method	Number of cases				Number of controls				P_HWE_
					GG	GA	AA	Total	GG	GA	AA	Total	
Gao et al.^*^	2021	China	Colorectal cancer	TaqMan	202	185	43	430	256	159	30	445	0.43
Shaker et al. (21)	2021	Egypt	Breast cancer	TaqMan	63	117		180	93	57		150	–
Kong et al. (22)	2020	China	Gastric cancer	TaqMan	215	198	61	474	290	203	50	543	0.1
Xu et al. (23)	2020	China	Prostate cancer	TaqMan	98	54	13	165	111	78	11	200	0.57
Zheng et al. (14)	2020	China	Breast cancer	MassArray	224	170	33	427	403	250	47	700	0.33
Ali et al. (24)	2020	Egypt	Breast cancer	TaqMan	57	63	30	150	84	63	7	154	0.26
Mazraeh et al. (25)	2020	Iran	Acute myeloid leukemia	PCR-based restriction fragment length polymorphism	43	36	21	100	16	48	36	100	1
Wei (26)	2019	China	Liver cancer	Taqman	717	349	51	1117	795	391	62	1248	0.13
Yang et al. (16)	2018	China	Lung cancer	Taqman	268	219	39	526	289	204	33	526	0.71
Zhuo et al. (27)	2018	China	Neuroblastoma	TaqMan	233	141	18	392	433	296	54	783	0.72
Zhang et al. (28)	2018	China	Gastric cancer	TaqMan	83	74	15	172	138	76	10	224	0.91
Cao et al. (29)	2016	China	Colorectal cancer	TaqMan	264	200	52	516	298	188	31	517	0.85

^*^Current study.

**Table 3:** Meta-analysis of the association between the rs7158663 polymorphism and cancer risk.

Comparison	^*^Subgroup	Heterogeneity		Effect model	OR[95%CI]	P
		P_H_	I^2^			
(AA+AG) vs. GG	Overall	<0.00001	83%	Random	1.22[0.99,1.49]	0.06
	China	0.003	65%	Random	1.18[1.02,1.37]	0.03
	Egypt	0.18	44%	Fixed	2.44[1.77,3.37]	<0.00001
	Breast cancer	0.001	85%	Random	1.89[1.08,3.30]	0.02
	Gastric cancer	0.36	0%	Fixed	1.47[1.19,1.81]	0.0004
	Colorectal cancer	0.38	0%	Fixed	1.40[1.17,1.68]	0.0003
AA vs. (AG+GG)	Overall	0.0003	70%	Random	1.27[0.95,1.70]	0.11
	China	0.11	39%	Fixed	1.23[1.05,1.44]	0.01
	Breast cancer	0.002	89%	Random	2.36[0.54,10.42]	0.26
	Gastric cancer	0.47	0%	Fixed	1.55[1.09,2.22]	0.02
	Colorectal cancer	0.7	0%	Fixed	1.65[1.18,2.31]	0.003
AA vs. GG	Overall	<0.00001	80%	Random	1.31[0.90,1.90]	0.16
	China	0.02	55%	Random	1.33[1.03,1.73]	0.03
	Breast cancer	0.002	90%	Random	2.70[0.55,13.13]	0.22
	Gastric cancer	0.39	0%	Fixed	1.78[1.23,2.58]	0.002
	Colorectal cancer	0.91	0%	Fixed	1.86[1.32,2.62]	0.0004
AG vs. GG	Overall	0.0004	69%	Random	1.11[0.94,1.31]	0.21
	China	0.04	51%	Random	1.15[1.01,1.31]	0.04
	Breast cancer	0.51	0%	Fixed	1.27[1.02,1.60]	0.04
	Gastric cancer	0.41	0%	Fixed	1.39[1.12,1.74]	0.003
	Colorectal cancer	0.29	10%	Fixed	1.32[1.09,1.60]	0.004
A vs. G	Overall	<0.00001	84%	Random	1.14[0.96,1.34]	0.14
	China	0.0007	70%	Random	1.16[1.02,1.32]	0.02
	Breast cancer	0.004	88%	Random	1.53[0.87,2.69]	0.14
	Gastric cancer	0.32	0%	Fixed	1.38[1.17,1.63]	0.0001
	Colorectal cancer	0.61	0%	Fixed	1.36[1.17,1.56]	<0.0001

^*^ Two and more studies were combined for analysis.

**Figure 3:**
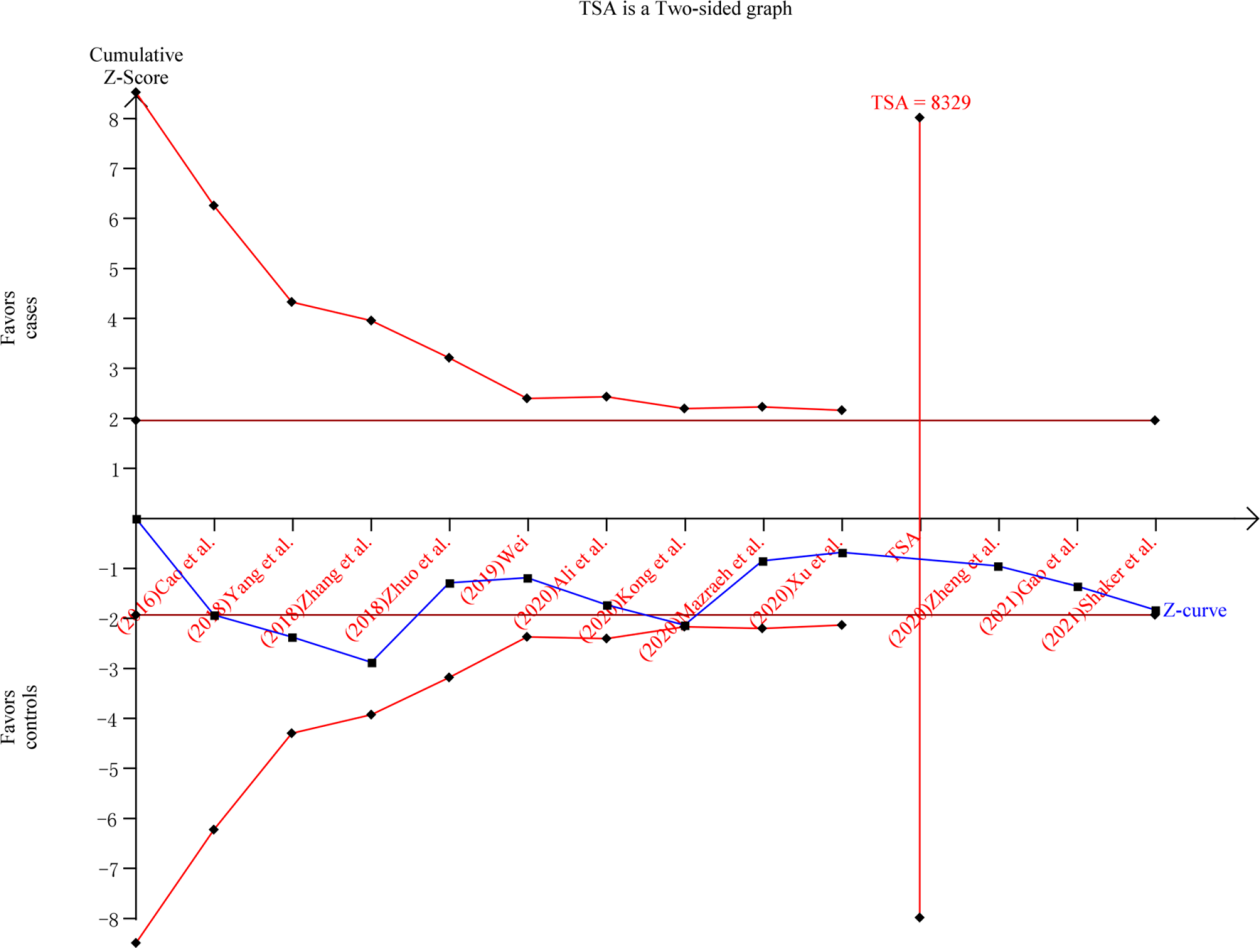
Trial sequential analysis of the current meta-analysis under the (AA+AG) vs. GG model.

The sensitivity analysis showed that after Mazraeh’s study was removed, the overall combined results were significantly altered under all comparison models (**Figure S2**). After Zhuo’s study was removed, the overall combined results were significantly altered under (AA+AG) vs. GG, and AA vs. (AG+GG) models. After Xu’s study was removed, the overall combined results were significantly altered under (AA+AG) vs. GG model. Begg’s and Egger’s tests showed no publication bias in the current meta-analysis (**Table 4**). TSA was conducted in the (AA+AG) vs. GG model. The result showed that the cumulative Z-curve (blue line) has crossed the required information sizes (n=8,329) (**Figure 3**), which indicated that the cumulative evidence was adequate in the overall analysis.

**Table 4:** Publication bias analysis of included studies.

Comparison	P value	
	Begg’s test	Egger’s test
(AA+AG) vs. GG	1	0.58
AA vs. (AG+GG)	0.53	0.57
AA vs. GG	0.64	0.39
AG vs. GG	0.76	0.60
A vs. G	0.64	0.39

## Discussion

Recent studies have shown that certain genetic variants on lncRNA genes may be associated with cancer risk (30–32). These genetic variants contain the rs7158663 polymorphism on the *MEG3* gene. For instance, Cao et al. found that rs7158663 AA genotype had significantly higher CRC risk than GG genotype, which was consistent with our results. The further stratified analysis revealed that the elevated risk was strongly associated with people with age ≤ 60 and a family history of cancer. However, there was no link found between the rs7158663 polymorphism and CRC site or stage (29). Both Zhang et al. and Kong et al. found that individuals carrying the rs7158663 AG+AA genotype or A allele had a significantly increased risk of gastric cancer (22, 28). However, some studies suggested that the rs7158663 polymorphism was not associated with cancer risk. For instance, Wei found that the rs7158663 polymorphism was not associated with hepatocarcinogenesis (26). Yang et al. found that the rs7158663 polymorphism was not associated with susceptibility to lung cancer (16). Zhuo et al. found that the rs7158663 polymorphism was not linked with neuroblastoma susceptibility, regardless of whether it was corrected for age and gender (27). These inconsistent results forced us to clarify the relationship between the rs7158663 polymorphism and cancer susceptibility by meta-analysis. By combining two and more studies for analysis, we found that the rs7158663 polymorphism was not associated with overall cancer susceptibility. However, the country-based stratified analysis showed that the rs7158663 polymorphism was associated with cancer susceptibility in the Chinese population under (AA+AG) vs. GG, AA vs. (AG+GG), AA vs. GG, AG vs. GG, and A vs. G models, and in the Egyptians under (AA+AG) vs. GG model. The stratified analysis based on cancer type showed that the rs7158663 polymorphism was associated with susceptibility to breast cancer under (AA+AG) vs. GG and AG vs. GG models, and gastric cancer under (AA+AG) vs. GG, AA vs. (AG+GG), AA vs. GG, AG vs. GG, and A vs. G models, and colorectal cancer under (AA+AG) vs. GG, AA vs. (AG+GG), AA vs. GG, AG vs. GG, and A vs. G models. There was no publication bias in the current meta-analysis, and TSA suggested that the sample size in the overall combined analysis was adequate. However, the sensitivity analysis results suggested that the current meta-analysis results were not sufficiently stable. Therefore we needed more studies to confirm the current findings.

MEG3 could inhibit the malignant phenotype of many cancers including gastric, breast and colorectal cancers (33–35). The current study found that the rs7158663 polymorphism could affect MEG3 expression in colorectal tissues. MEG3 expression was significantly lower in colorectal tissues of the AA or GA genotype compared to those of the rs7158663 GG genotype. Bioinformatics analysis showed that the rs7158663 A allele contributed to the binding of hsa-miR-4307 and hsa-miR-1265 to MEG3. Therefore, we speculated that the rs7158663 polymorphism may affect an individual’s susceptibility to CRC by influencing the regulation of MEG3 expression by miRNAs.

Although the current study has yielded some meaningful results, some shortcomings needed to be pointed out. Due to the insufficient sample size of the current case-control study and the unavailability of some clinical data, we did not further analyze the relationship between the rs7158663 polymorphism and the clinicopathological features. In addition, we did not consider the effect of the rs7158663 polymorphism interaction with environmental factors on cancer susceptibility.

## Conclusions

The current study results suggest that the *MEG3* gene rs7158663 polymorphism is associated with susceptibility to a variety of cancers, such as breast cancer, gastric cancer and CRC. However, large and well-designed studies are still needed to validate our findings.

## Data Availability Statement

The original contributions presented in the study are included in the article/**Supplementary Material**. Further inquiries can be directed to the corresponding author.

## Ethics Statement

The studies involving human participants were reviewed and approved by Shanghai Xuhui District Central Hospital. The patients/participants provided their written informed consent to participate in this study.

## Author Contributions

XG carried out the molecular genetic studies, did the literature search and the statistical analysis, and wrote the paper. XL performed biochemistry tests. XW were responsible for the acquisition of data. SZ participated in study design and coordination and helped to draft the manuscript. XG and SZ interpreted the data and were responsible for the manuscript preparation. All authors contributed to the article and approved the submitted version.

## Conflict of Interest

The authors declare that the research was conducted in the absence of any commercial or financial relationships that could be construed as a potential conflict of interest.

## Publisher’s Note

All claims expressed in this article are solely those of the authors and do not necessarily represent those of their affiliated organizations, or those of the publisher, the editors and the reviewers. Any product that may be evaluated in this article, or claim that may be made by its manufacturer, is not guaranteed or endorsed by the publisher.
