# Supplementary material for: The Association of MEG3 Gene rs7158663 Polymorphism With Cancer Susceptibility
Source: Front Oncol. 2021 Dec 9;11:796774. doi: 10.3389/fonc.2021.796774 (PMC8695896; doi:10.3389/fonc.2021.796774)
Supplement: Supplementary file 1 [file DataSheet_1.doc]

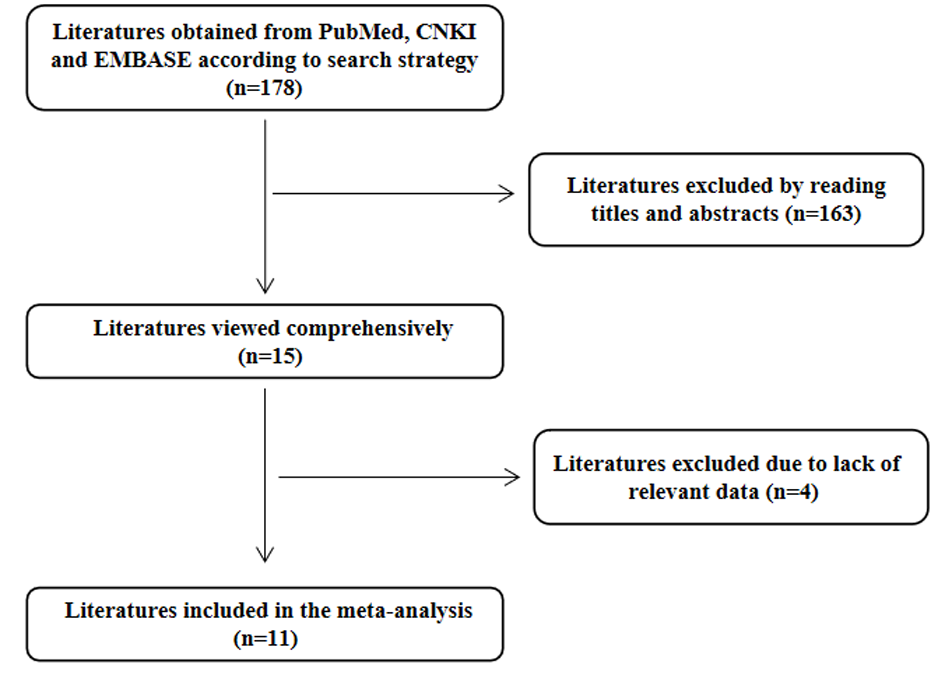


Figure S1. Flow chart of meta-analysis study selection


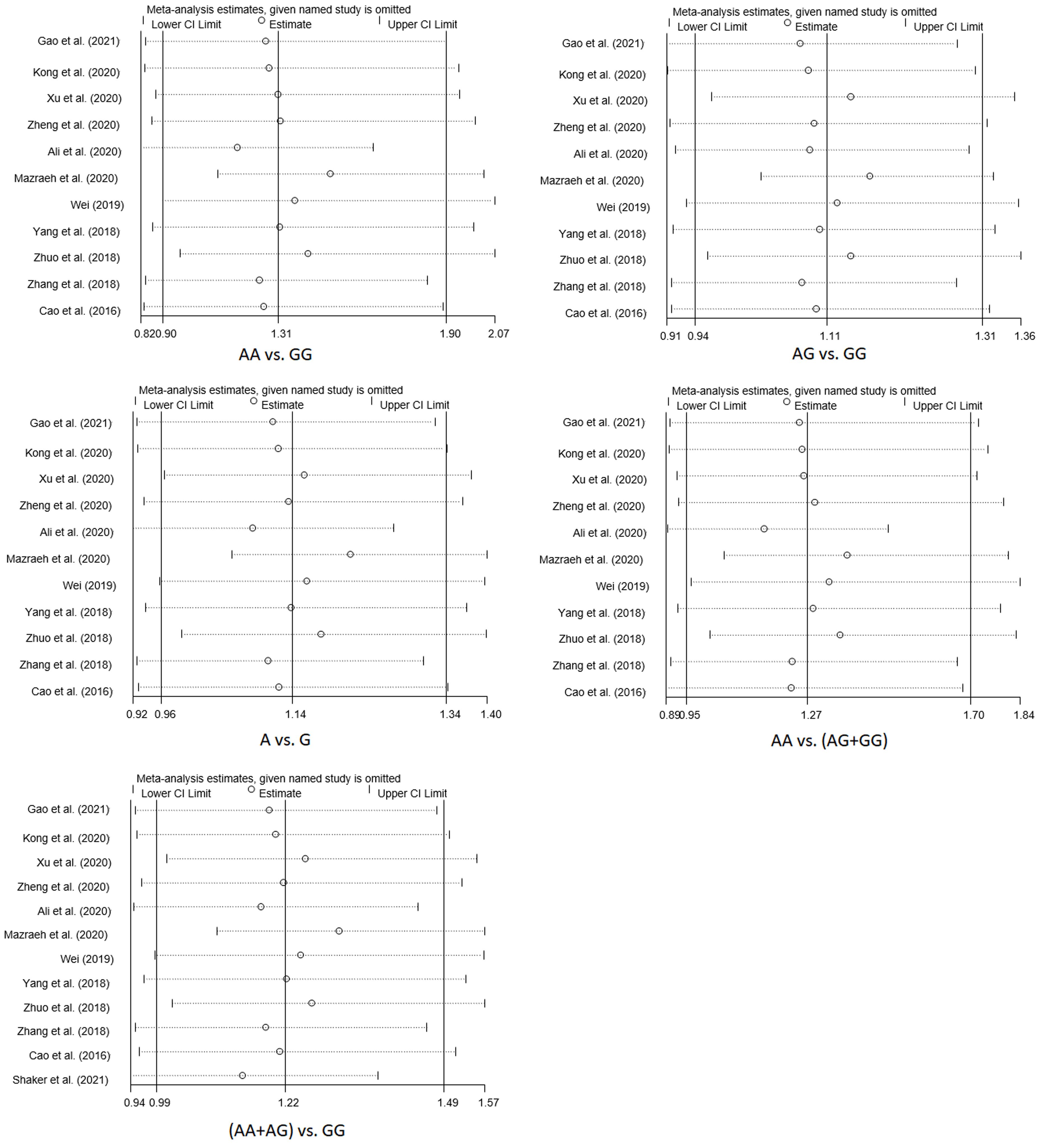


Figure S2. Sensitivity analysis of the overall result

**Table S1. The specific primer sequences for quantitative real-time PCR**

| Genes | Primer sequences |
| --- | --- |
| MEG3 | Forward: 5′-GCCATCACCTGGATGCCTACG-3′ |
|  | Reverse: 5′-TGGGAATAGGTGCAGGGTGTC-3′ |
| GAPDH | Forward: 5′-GTCTCCTCTGACTTCAACA-3′ |
|  | Reverse: 5′-TGAGGGTCTCTCTCTTCCT-3′ |
